# Personal

**Published:** 1905-01

**Authors:** 


### PERSONAL.

---

Dr. James Wright Putnam, of Buffalo, sailed from Baltimore, December 28, 1904, on the steamer Athos, bound for Panama, where he will attend the Fourth Pan-American Medical Congress. He will also attend the meeting of the American Public Health Association the following week at Havana. He is accompanied by Mrs. Putnam, and will be absent from home about four weeks. Dr. Putnam will read a paper at the Panama congress entitled, Paranoia as related to homicide.

---

Dr. F. M. Gipple, of Bomansville, near Buffalo, sustained the loss of his residence and barns by fire November 26, 1904. The fire occurred about 3 o'clock in the morning, affording the doctor and his family scant opportunity to escape. His wife and four daughters, however, were unharmed, but Dr. Gipple, himself, sustained severe burns of the hands and feet while rescuing his family.

---

Dr. A. W. Jayne, of North Tonawanda, N. Y., has been appointed health physician of that city to fill the term made vacant by the removal of his predecessor, Dr. Leonhardt, to Porto Rico.

---

Dr. Frank P. Foster, editor of the *New York Medical Journal*, and who, by the retirement of Dr. George F. Shrady from the editorial field, became the dean of the medical editorial corps in the United States, celebrated the twenty-fifth anniversary of his editorship of the *New York Medical Journal*, December 31, 1904. One of the special features of the day was a luncheon given to Dr. Foster at the Hardware Club, where many of his professional and other friends assembled to do honor to the guest and to accentuate the interesting occasion by clever speech and witty story.

---

Dr. Charles H. North, formerly of Buffalo and a graduate of the University of Buffalo in 1898, has been promoted from assistant physician to superintendent of the Dannemora state hospital.

---

Dr. Charles E. Congdon, of Buffalo, who has been afflicted with gallstone disease for some time, manifested by recurrent attacks of biliary colic, was operated upon by Dr. Joseph Price, of Philadelphia, December 5, 1904, at Dr. Congdon's hospital. He is now convalescent and soon expects to resume his professional work.

---

Dr. Walter D. Greene, health commissioner of Buffalo, attended the fourth annual convention of sanitary officers of the state of

New York, which was held recently at Albany. The meeting was attended by about 250 public health officials, before whom Dr. Greene read a paper entitled, Legal methods and forms of procedure in sanitary work.

---

Dr. Edwin Ricketts, of Cincinnati, was recently operated upon at the Good Samaritan Annex for umbilical hernia by Dr. Charles A. L. Reed, of that city. His convalescence was prompt and his recovery complete.

---

Dr. George W. Crile, of Cleveland, was entertained at dinner by Dr. Roswell Park, Tuesday evening, December 6, 1904. Dr. Crile read a paper the same evening before the section on surgery of the Buffalo academy of medicine entitled, Prevention and treatment of shock and hemorrhage in surgical practice.

